# 

**Published:** 1898-02-26

**Authors:** 


					Vhu aoiiPifAL^m n The standardio^highest purity." ?Lanceti*'EB'.M:2(jTHl"l893r
CADBURY'S     AAnDI,DV"
COCOA
COCOA / V\6B/
ABSOLUTELY PURE. i ^; NO DRUGS OR ALKALIES USED, *3?
pM, o d
WMWH HOSPITAL
No. .596'. Vol, XXIII. raegj Sattjedat,
Feb. 26th, 1898.
[Subscription Tema.] pRICE TWOPENCE

				

